# Tensile Examination and Strength Evaluation of Latewood in Japanese Cedar

**DOI:** 10.3390/ma15072347

**Published:** 2022-03-22

**Authors:** Akihiro Takahashi, Naoyuki Yamamoto, Yu Ooka, Toshinobu Toyohiro

**Affiliations:** 1 Department of Mechanical Engineering, National Institute of Technology, Miyakonojo College, 473-1 Yoshio, Miyazaki 885-8567, Japan; naogen@cc.miyakonojo-nct.ac.jp (N.Y.); toyohiro@cc.miyakonojo-nct.ac.jp (T.T.); 2 Architecture, National Institute of Technology, Miyakonojo College, 473-1 Yoshio, Miyazaki 885-8567, Japan; y-ooka@cc.miyakonojo-nct.ac.jp

**Keywords:** Japanese cedar, latewood, mechanical property, fracture surface observation

## Abstract

With the crisis awareness of global warming and natural disasters, utilization of local wood has drawn increasing attention in achieving the Sustainable Development Goals (SDGs). It is necessary to investigate the deformation and fracture of the structural tissue in wood in order to improve the safety and reliability of wood application. However, deformation and fracture mechanisms of the structural tissue in each annual ring are unknown. The mechanical characteristics of wood are reflected in the properties of earlywood and latewood. In the present study, microstructural observation and tensile tests were conducted to examine the relationship between the mechanical properties and fracture behavior of latewood in the growth direction in Japanese cedar. Brittle fracture behavior of the latewood specimen was confirmed based on the tensile stress–strain curve and features of the fracture surface. Moreover, two fracture modes, tensile fracture and shear fracture, were recognized. Weibull analysis of tensile strength in each fracture mode was performed to evaluate the reliability and utility of brittle latewood. Lastly, two fracture mechanisms were discussed based on the failure observation findings by a scanning electron microscope.

## 1. Introduction

The increasing demand for materials made from renewable sources with a small environmental load has increased recently. This is due to several driving forces such as shortages of natural resources, changes in consumers and their concerns over social environmental issues, and the SDGs [1]. As a naturally grown material with carbon sequestration properties, wood has significant appeal as a sustainable material. The use of wood in industry and construction can reduce carbon in nature [2]. Therefore, wood is an environmentally friendly material that has been used for the construction of houses [3], marine environments [4], bridges [5,6,7], and wooden goods for many centuries. Regardless of species, engineered wood is a valuable construction material because of their highly desirable strength/density index. Significant progress in technology has been made for the last several decades to push the limit of wood construction with an advantage of higher strength/density index than other materials. As a result, there has been a noteworthy shift in public perception in terms of the acceptance of wood as a material for high-rise buildings by engineered wood such as glued laminated timber, GLT laminated veneer lumber, LVL, and cross-laminated timber, CLT [8]. There is already a growing list of high-rise wooden buildings that have been constructed in different countries [9], and the trend is expected to continue. In general, buildings up to 10 stories tend to use the CLT as the primary structure [10,11,12,13,14,15]. The longitudinal elastic modulus and tensile strength of the GLT beam and CLT wall panel are one of the important characteristic values that determine the suitability of the high-rise buildings.

**Figure 1:**
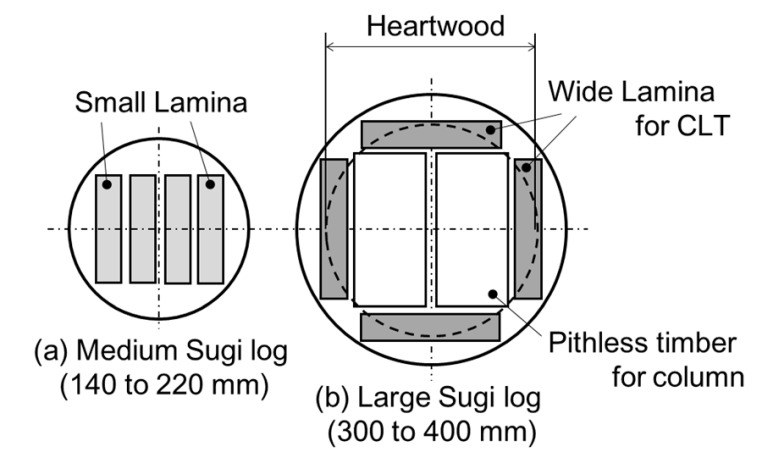
Cutout arrangement of lamina from different log diameters.

Japanese cedar (*Cryptomeria japonica*) called Sugi, a kind of conifer, is the most produced wood in Japan, accounting for 57% in 2017 [15] and is expected as domestic lamina in all layers for the GLT and CLT frames [16] in Japan. The main supply of raw Sugi tree is shifting from medium-diameter logs (with a diameter between 140 and 220 mm) to large-diameter logs due to Japanese government’s policies with respect to forestry management, and the production of large logs (over 300 mm in diameter) is increasing significantly within the timber manufacturing industry [17]. In an earthquake-prone country such as Japan, anti-seismic buildings with large-sized CLT are desired. For that reason, facilitating the collection of wide laminae from outside in a large-diameter log for large-sized laminated timbers is efficient, as shown in Figure 1.

Although some information on Sugi timber quality has been obtained for medium Sugi logs, little is known about the quality in the large logs, the supply of which is expected to increase imminently. Studies have been carried out to evaluate mechanical properties of engineered woods such as GLT and CLT made of medium Sugi logs [18,19,20,21,22] or large Sugi logs [23,24,25,26,27] by Japanese researchers. According to research by Ido et al. [19] using lamina taken from large Sugi logs, the estimated tensile strength of CLT, as calculated using the Young’s modulus of lamina of each layer, and the tensile strength of a lamina unit were found to be in good agreement with the measured tensile strength of CLT. On the other hand, the modulus of elasticity, MOE, and the modulus of rupture, MOR, in a Sugi sample by bending tests have widely been researched related to wood properties such as density, moisture content, microfibril angle (MFA), and so on [28,29,30,31,32,33,34], where these characteristics in medium-diameter Sugi logs were examined. However, tensile properties in the growth direction with respect to the microtome sample in the outer region where the lamina is cut have not been investigated yet. Wood is a hierarchically structured material with levels that might be termed as the tree structure, macroscopic (annual rings), microscopic (cellular), ultrastructural (cell walls), and biochemical levels (polymers such as cellulose, hemicellulose, and lignin). Thus, to utilize the functions of large-diameter Sugi logs, knowledge of their physical and mechanical properties is necessary as a whole. As above, for the engineered wood structure design such as large-sized GLT and CLT, knowledge of wood strength and rigidity is fundamental. However, previous studies have mostly focused on wood properties related to macro cross-sectional characteristics. Considering the current state of knowledge, it is a truism to claim that the mechanical strength of a wood material depends on its microstructure. Moreover, the microscopic mechanisms underlying the mechanical performance of wood need to be explained to meet the wood demand and improve technical skills and structural design technologies. To this end, in-depth studies on large-diameter Sugi logs for CLT have just begun.

**Figure 2:**
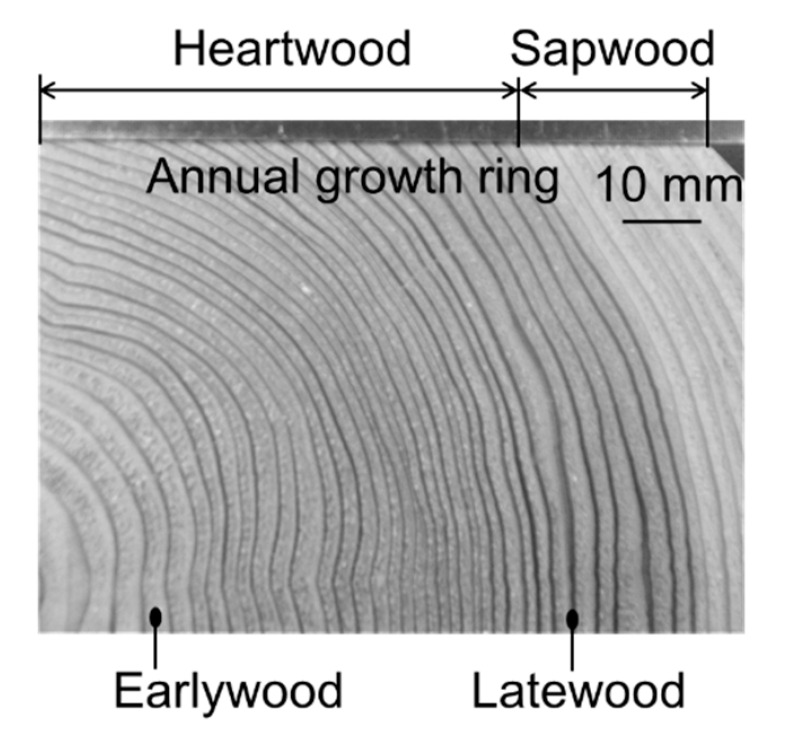
Annual growth ring in Japanese cedar used in present study.

An annual growth ring in Japanese cedar is shown in Figure 2. In general, latewood and earlywood are determined based on the plane cut of a tree log. The latewood region has a narrow wall thickness of 0.1 to 0.4 mm.

At present, there are no material testing standards for tensile tests using miniature wood test pieces. However, a suitable tensile examination method for thin latewood in Japanese cedar was previously proposed in [35]. Moreover, it was clarified that the failure behavior of latewood follows two macroscopic fracture patterns [36]. However, their fracture mechanisms remain unclear. Fewer studies have shown the tensile properties and fracture behavior in latewood taken from the outer region in large-diameter Sugi logs and to accumulate fundamental data on wood properties. The aim of the present study is to investigate the tensile stress–strain behavior of a latewood as a simple substance specimen collected from the outer side in heartwood in a Sugi log. In addition, Weibull analysis is performed on the fracture strength of latewood, and different failure behaviors of the latewood are discussed based on experimental results in SEM observation. There is a lack of research into learning the from microtome sector to surely understand the tensile behavior in latewood. The present study will improve our knowledge and skills of holistic wood utilization and structural design techniques on manufacturing engineered woods such as large-sized GLT and CLT by Japanese cedar.

## 2. Materials and Methods

### 2.1. Experimental Material

Wood samples in this study were cedar from Nichinan city in southern Kyushu, Japan. The tree age was 40 years. The cut log had a body diameter of 300 mm (up to 4 m above the ground) and was sun-dried. The moisture content (*MC*) defined as the weight of water in the cut log was given as a percentage of the oven-dried weight [37]:

(1) $$MC=\frac{moist\;weight-oven-dried}{oven-dried}\times 100\left(\%\right),$$

The *MC* in the cut log was 12%. The wood density calculated from the green volume and air-dry weight was 370 kg/m^3^ [37].

**Figure 3:**
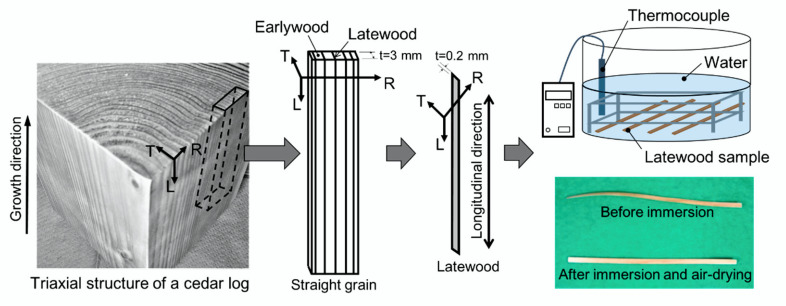
Manufacturing process and orientation of the latewood tensile specimen.

Latewood samples were collected from rings 25 to 35 starting from the pith (i.e., outside heartwood). The manufacturing process and water immersion of the latewood tensile specimens in the growth direction taken from the cut log is shown in Figure 3. The specimen for tensile testing was cut in a straight grain orientation using a cutter tool and sandpaper. After cutting, the specimen was immersed in water for 24 h to remove residual strain on the specimen after cutting. After immersion, the specimen was dried in ambient air (relative humidity of 65% and room temperature of 298 K) for 72 h. Based on [38,39,40], the specimen thickness (radial) was 0.2 ± 0.05 mm, width (tangential) was 3.0 ± 0.5 mm, and length (longitudinal) was 130 mm.

**Figure 4:**
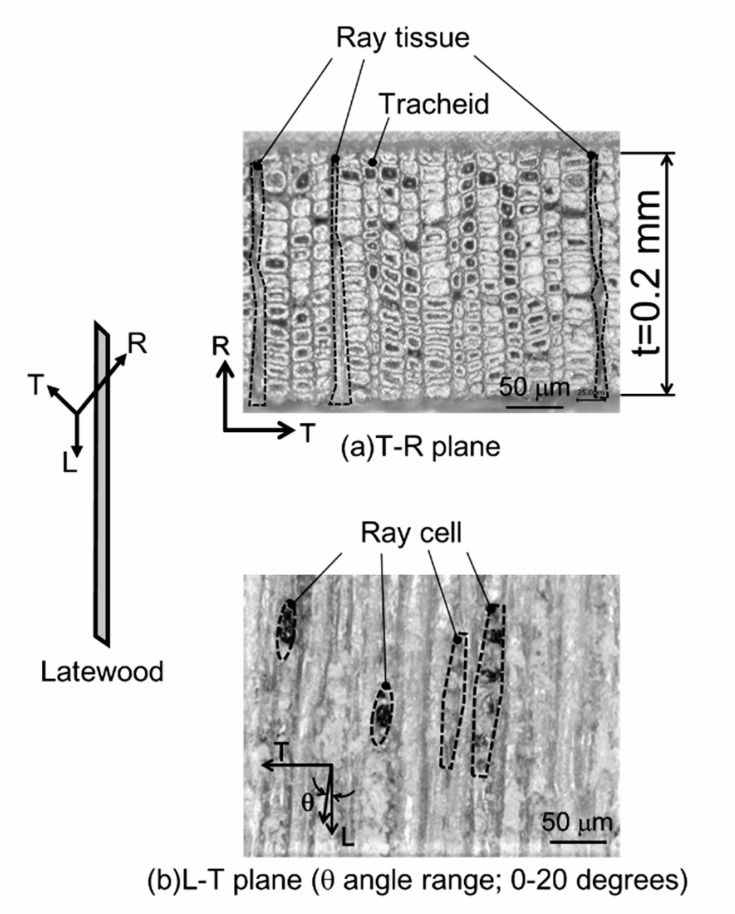
Microstructures in the two orientations in the latewood specimen: T-R plane (**a**) and L-T plane (**b**).

Microstructures of the latewood specimen in two orientations, T-R plane (a) and L-T plane (b), are shown in Figure 4. Tracheids with square or slightly rectangular cells of differing thickness were observed in the T-R plane. The thickness of the cell wall of tracheids ranged from 1 to 5 μm. The other region (three broken-line frames) running from the top to the bottom of the microstructure was the ray tissue. The tracheids and ray cells had a specific tilt angle, *θ* to the L direction in the L-T plane (b). The range was approximately 0 to 20°, measured by a protractor qualitatively. The density and *MC* in the latewood specimen measured by [37] were 897 kg/m^3^ and 10%, respectively.

### 2.2. Tensile Examination Procedure

**Figure 5:**
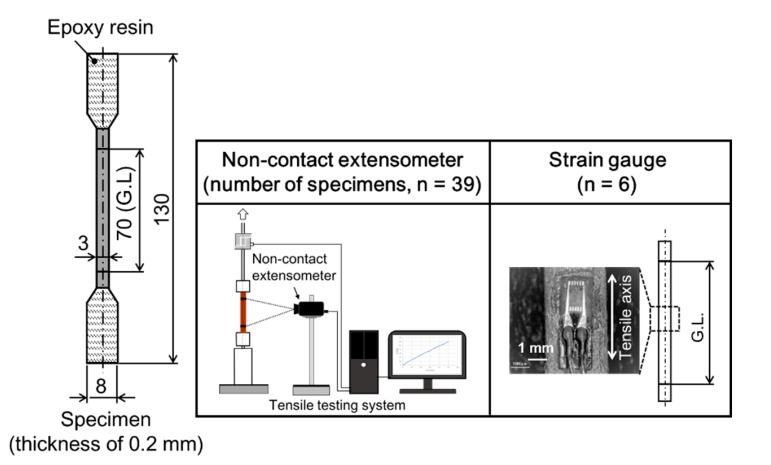
The experimental information e in the present study.

**Figure 6:**
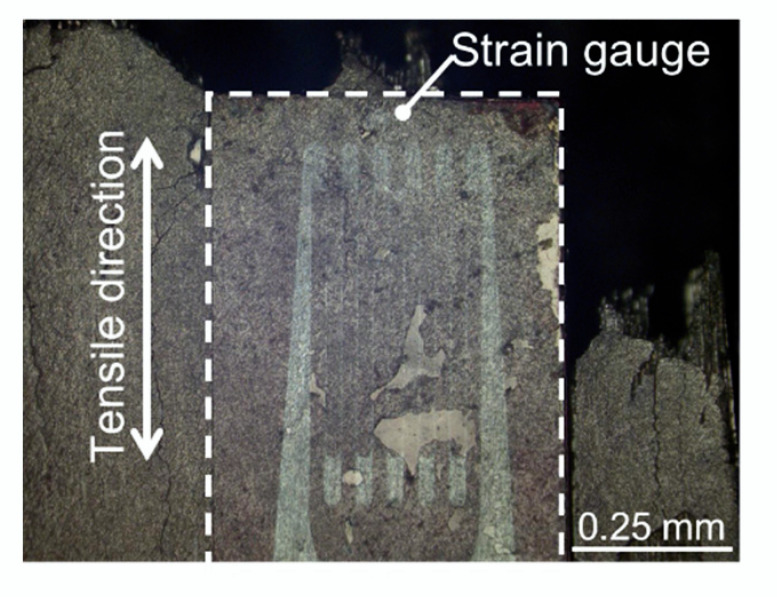
Failure along the edge of the strain gauge occurred during tensile testing.

Tensile testing of the latewood specimen was performed in accordance with Japanese industrial standards, JIS Z2241 [41], at an initial loading speed of 1.0 mm/min. The universal testing system (EZ-SX, Shimadzu) was used to assess mechanical properties in the present study. Tensile load, *F*, was measured by a one-side loaded cell with a capacity of 500 N, and displacement and tensile strain of the tensile specimen in the growth direction were measured using a noncontact extensometer (DVE-101/201, Shimadzu, Kyoto, Japan) and one-side strain gauge (FLK-1-11, Tokyo Measuring Instruments Lab., Tokyo, Japan) [42]. For area measurement to calculate tensile stress, *σ*, and elastic modulus, *E*, the average value of three measured cross-sectional areas, *A*, of the specimen in the longitudinal axis before tensile examination was used. Therefore, engineering tensile stress, *σ = F/A*. *E*, was determined as a proportion of a regression line fitted to the stress–strain chart between 10 and 30 MPa. The 6 specimens attached to the strain gauge were turned out and 39 specimens without the gauge were prepared for tensile examination using a noncontact type extensometer, as shown in Figure 5. In advance, a tensile test was conducted using a thin metallic lead (*Pb*, thickness of 0.2 mm and elastic modulus of 16 GPa) attached to a strain gauge, and it was confirmed that the elastic modulus was measurable without the influence of the adhesive. A microphotograph of the specimen that failed along the frame of the attached strain gauge is shown in Figure 6. This failure was considered to be due to the hardness of the adhesive. As a result, the deformation in the elastic region was evaluated by a strain gauge and the noncontact type extensometer was used for fracture strain evaluation.

An optical microscope (OM, VHX-2000, KEYENCE, Osaka, Japan) and electrical scanning microscopy (SEM) were used to observe the microstructural features. SEM (S-4800, Hitachi High-Technologies Corp., Ibaraki, Japan) was also used to examine the fractographic features. For interior views of the SEM observation, sputter coating with the Pt-Pd target was conducted.

### 2.3. Tensile Strength Evaluation by Weibull Statistics

The Weibull analysis was employed to assess a wide range of issues, including the mechanical properties of brittle materials and life-time testing [43]. The two-parameter continuous probability density function for tensile strength variables is expressed by:

(2) $$P=\left(\frac{m}{\sigma_{0}}\right){\left(\frac{\sigma }{\sigma_{0}}\right)}^{m-1}exp\left[-{\left(\frac{\sigma }{\sigma_{0}}\right)}^{m}\right],$$

The mean density function is asymmetrical and will assume only positive values. The symbol *m* is the Weibull modulus and *σ_0_* is the scale parameter.

The cumulative distribution function that gives the probability of failure *P* at stress *σ* is expressed as:

(3) $$P=1-exp\left[-{\left(\frac{\sigma }{\sigma_{0}}\right)}^{m}\right],$$

where *P* is the probability of failure at stress, *σ_B_*. In this study, the tensile strength of latewood was assessed by Weibull analysis.

## 3. Results and Discussion

**Figure 7:**
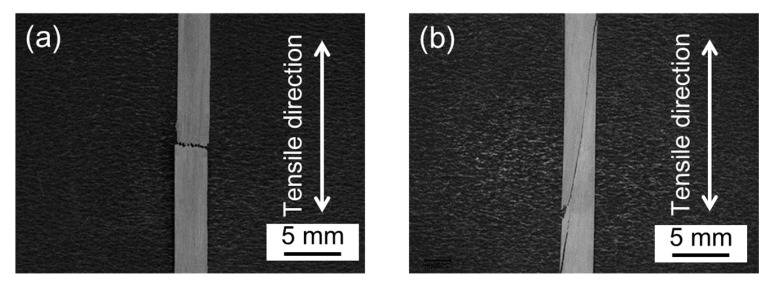
Classified failure modes were tensile fracture (**a**) and shear fracture (**b**).

The two dominant fracture modes observed in the present study, tensile fracture (a) and shear fracture (b), are shown in Figure 7. The macroscopic crack path of tensile fracture was perpendicular to the loading direction. On the other hand, the shear crack grew across to both ends of the specimen, causing a shear fracture, (b). In addition, the specimen with a *θ* of 0 to 11° exhibited a tensile fracture and another *θ* of 12 to 20° exhibited a shear fracture. This suggested a strong relationship between the *θ* and the fracture behavior of latewood.

**Figure 8:**
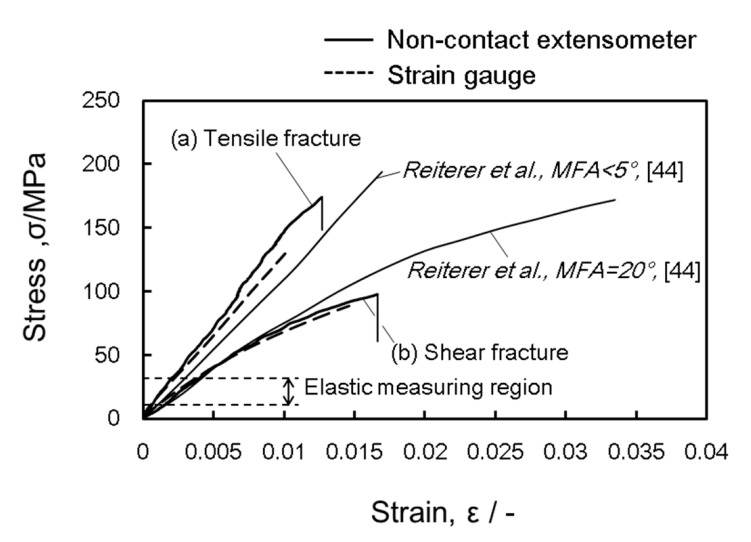
Stress–strain curves obtained from tensile examination. The curves correspond to the two respective failure modes.

Tensile stress–strain curves, which correspond to the two fracture modes, are shown in Figure 8. The curve of the tensile fracture was an approximately linear mechanical response, whereas that of the shear fracture followed a gentle curve from the elastic region. The applied stress was at a maximum (i.e., tensile strength, *σ_B_*) and the tensile specimen quickly broke. This brittle fracture behavior also occurred at a low loading speed of 0.01 mm/min. Reiterer et al. reported that stress–strain curves of a latewood tensile specimen with MFA <5° and =20° in *Prica abies* [44]. Results on the fracture observation photograph for both were unknown in [44], but it is interesting that they were close to those in our experimental findings. The measurement of MFA in testing latewood was our next research task.

Several procedures have been suggested to determine Weibull parameters such as linear regression (the Weibull plot), weighted linear regression, and maximum likelihood [45,46,47]. The Weibull plot is the most common and simplest method to determine Weibull parameters. The tensile strength values are ranked from the minimum to the maximum and each value is assigned a probability of failure (*P*) based on its ranking, *i*, with *i* ranging from 1 to *n*, where *n* is the number of measurements of (tensile fracture, *n* = 19, and shear fracture, *n* = 20). The cumulative probability of failure (*P*) is calculated using the following equation:

(4) $$P_{i}=\frac{i-0.3}{n+0.4},$$

where *i* is the rank and *n* is the total number of data. In this study, *n* was the total number of data for the measured tensile strength.

**Figure 9:**
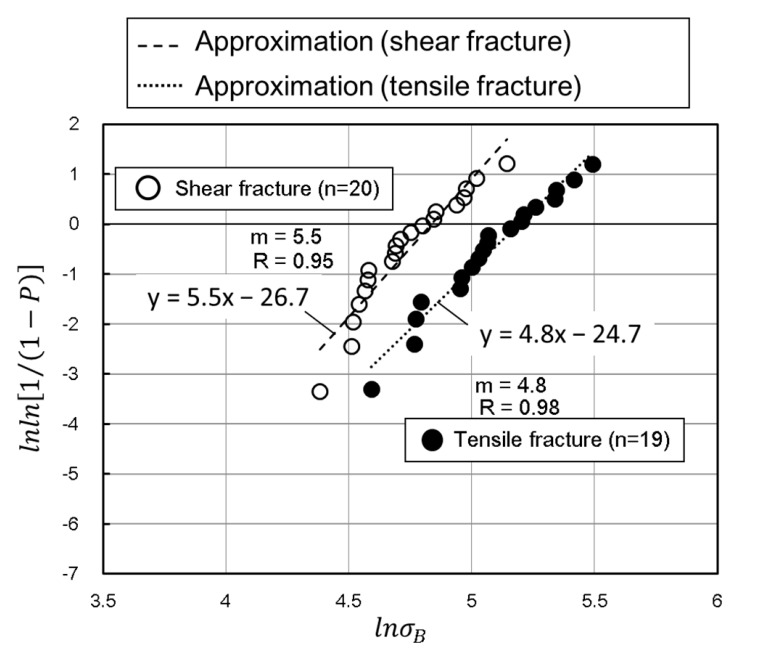
Weibull analysis plots corresponding to the two failure modes.

The results of Weibull analysis by plotting *lnln(1/1−P)* versus *lnσ_B_* for the tensile strength of the latewood specimen in the growth direction are shown in Figure 9. In the case, Equation (3) can be rearranged to give the following equation:

(5) $$lnln\left(\frac{1}{1-P}\right)=mln\sigma -mln\sigma_{0},$$

and the experimental tensile strength data plotted in Equation (5) give an approximate straight line from whose equation the parameters *m* and *σ_0_* can be estimated based on the linear regression. The Weibull parameter *m* of a tensile fracture was *m* = 4.8, with a correlation coefficient of *R* = 0.98, whereas that of a shear fracture was *m* = 5.5, with an *R* = 0.95. The mean tensile strength *σ_mean_* can be expressed as [48]:

(6) $$\sigma_{mean}=\sigma_{0}\Gamma \left(1+\frac{1}{m}\right),$$

where Γ(·) is a gamma function. The mean tensile strength *σ_mean_* calculated by Equation (6) in the shear fractured specimen was approximately 29% lower than that in the tensile-fractured specimen. Weibull parameters and other mechanical properties are summarized in Table 1.

The simple composite mechanics rule that can be utilized to take into account the tracheids’ orientation is the maximum stress criterion, assuming that the plane is modeled as a thin mat with fibers (analogous to the tracheids) oriented at an angle *θ* (analogous to the angle *θ* in Figure 4) to the fiber axis. Failure of the mat occurs either at a critical (local) stress value $\sigma_{1}\geq \sigma_{1u}$ parallel to the fibers, $\sigma_{2}\geq \sigma_{2u}$ perpendicular to the fibers, or at a shear stress $\tau_{12}\geq \tau_{12u}$ along the fibers. The (local) in-plane stresses working parallel and perpendicular to the fibers $\left(\sigma_{1},\;\sigma_{2},\;\tau_{12}\right)$ can then be expressed as the (global) stresses applied in the x- and y-directions of the fiber mat $\left(\sigma_{x},\;\sigma_{y},\;\tau_{xy}\right)$ according to [49]:

(7) $$\left\{\begin{array}{c} \sigma_{1}\\ \sigma_{2}\\ \tau_{12} \end{array}\right\}=\left[T\right]\left\{\begin{array}{c} \sigma_{x}\\ \sigma_{y}\\ \tau_{xy} \end{array}\right\},$$

where the transformation matrix is given by:

(8) $$\left[T\right]=\left[\begin{array}{ccc} cos^{2}\theta & sin^{2}\theta & 2cos\theta sin\theta \\ sin^{2}\theta & cos^{2}\theta & -2cos\theta sin\theta \\ -cos\theta sin\theta & cos\theta sin\theta & cos^{2}\theta -sin^{2}\theta \end{array}\right],$$

**Figure 10:**
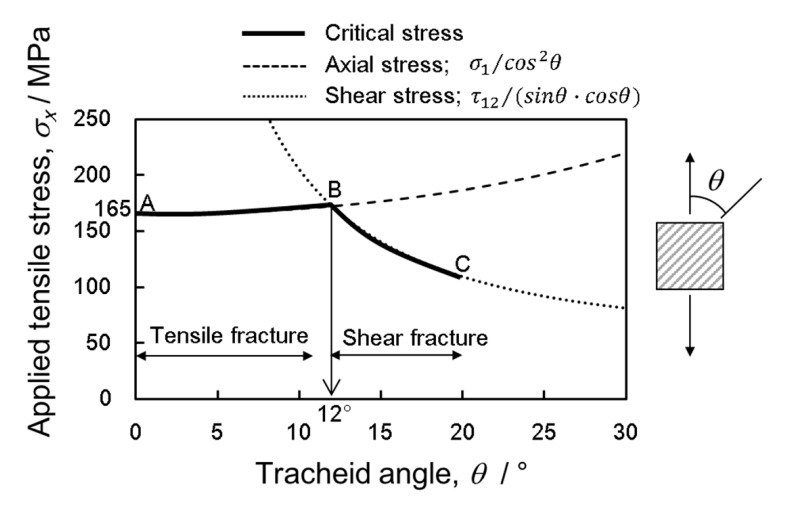
Predicted critical tensile strength depending on angle, *θ*, of the applied stresses for the onset of two failure modes.

If uniaxial tension $\left(\;\sigma_{2}=\tau_{12}=0\right)$ is assumed, the stress $\sigma_{xu}$ (i.e., applied tensile stress) to cause failure in the material can be expressed for each of the three failure modes as:

(9) $$\sigma_{xu}=\frac{\sigma_{1u}}{cos^{2}\theta },$$

(10) $$\sigma_{xu}=\frac{\sigma_{2u}}{sin^{2}\theta },$$

(11) $$\sigma_{xu}=\frac{\tau_{12u}}{cos\theta sin\theta },$$

where $\sigma_{xu}$ in Equations (9)–(11) indicate axial stress, transverse stress, and shear stress, respectively. Under the assumption of independent modes of failure with no interaction between each other and using experimental data for $\sigma_{1u}$, $\sigma_{2u}$, and $\tau_{12u}$, Equations (9)–(11) can be used to calculate the maximum tensile strength of latewood material with tracheids oriented at a given angle, *θ*. Conversely, data of the maximum (global) tensile strength can be predicted together with information on *θ* to calculate the local properties $\sigma_{1u}$, $\sigma_{2u}$, and $\tau_{12u}$, for a material. As shown in Figure 10, the predicted critical tensile strength for tensile fracture (solid line, AB) is given by

(12) $$\sigma_{ux}=\frac{165}{cos^{2}\theta };\;\left(0{}^{\circ}\leq \theta <12{}^{\circ}\right),$$

σ_mean_ is 165 MPa when indicating tensile fracture, and the angle, *θ =* 12°, is a specific tilt angle when transiting from tensile fracture to shear fracture. The two dashed lines of Equations (9) and (10) cross at point B. The stress value at point B is obtained at 173 MPa from Equation (12). Next, the shear fracture value,$\;\tau_{12u}$ at point B can be calculated by substituting $\sigma_{ux}=173\;\mathrm{MPa}$ in Equation (11), and a certain value, $\tau_{12u}=35\;\mathrm{MPa}$, is determined. Therefore, the predicted critical tensile strength for shear fracture (solid line, BC) for angles from 12 to 20° can be drawn, as shown in Figure 10. Further, it can be seen that Figure 10 qualitatively corresponds to tensile strength depending on the angle of the tracheid and therewith the prediction of the fracture behavior.

**Figure 11:**
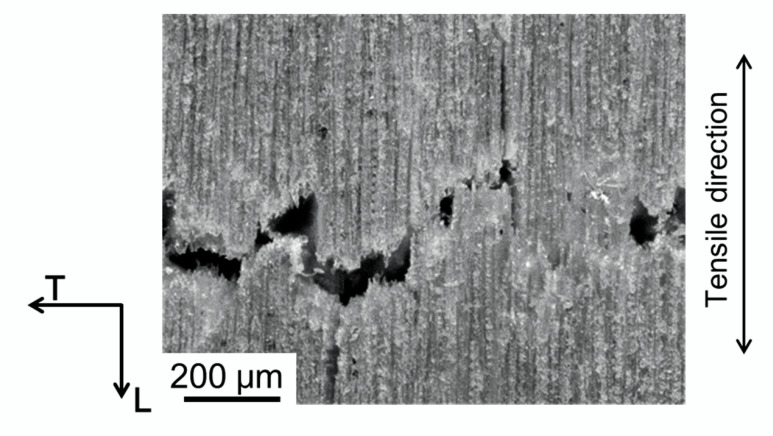
Serrated crack path in the tensile fracture observed in the L-T plane.

**Figure 12:**
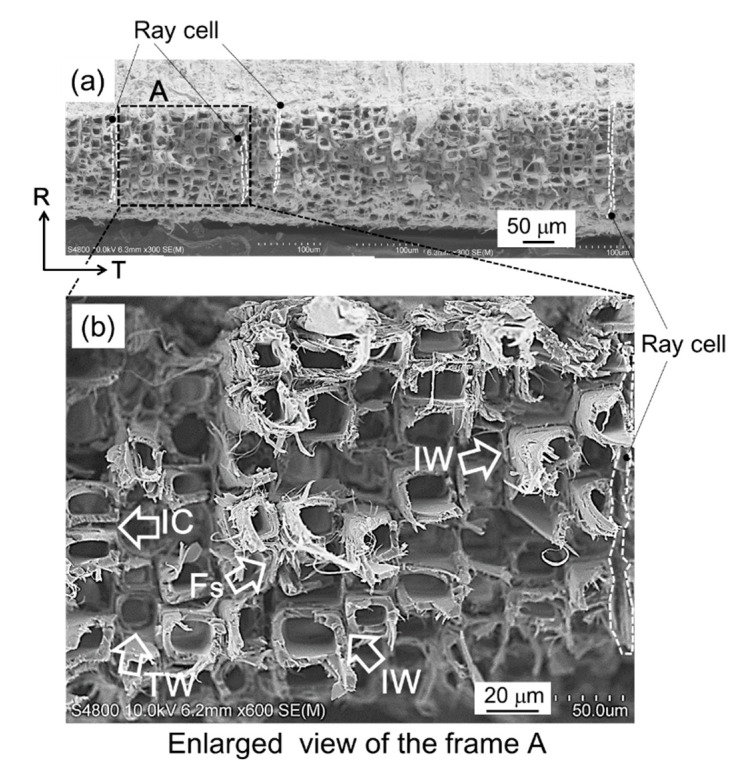
SEM image of the T-R fracture surface indicating the behavior of the tensile fracture. The enlarged view (**b**) is section A surrounded by the dashed lines in (**a**).

A photograph of the nearby crack path in the tensile fracture observed from the L-T plane is shown in Figure 11. Microscopic crack deflection was frequent during fracture propagation of the tensile fracture. The fracture surface observed from above is shown in Figure 12. Fracture modes of tracheids were observed, and the enlarged view of frame A shows fibrils in the tip of the fractured tracheid (Fs indicated by an arrow), the separation of cells at the middle lamella (IC indicated by an arrow), a crack path cut through the tracheids (TW indicated by an arrow), and an hierarchical crack propagation within the cell wall (IW indicated by two arrows). According to Côté and Hanna [50], three kinds of cell fractures in many species are recognized: intercell failure (IC), transwall failure (TW), and intrawall failure (IW). Intercell failure takes place at the middle tracheid lamella and is simply the interfacial debonding between tracheids at these junctions. Transwall failure is the complete rupture when the fracture path cuts across the wall. Intrawall failure occurs within the secondary wall and, in most instances, it is at the S_1_/S_2_ interface or close to it. These fracture characteristics were confirmed in the fracture surface in the tensile fracture. These fracture modes tended to produce a highly rough fracture surface, as shown in Figure 11. Figure 12 also shows fracture of the ray cell observed on the tensile fracture surface. It is well known that the structure and distribution of the ray cell have a strong relationship with the compressive mechanical property and its fracture behavior in Sugi timber [51]. In present study, the influence of the ray cell on the tensile fracture behavior is unknown. However, it is suggested that the tensile fracture is partly related because the fracture of the ray cell was included in a part of the crack path.

Ifju et al. [52] and Kifetew et al. [53] also observed fractures in the latewood region where the fibrils appeared at the tips of tracheids, as shown in Figure 12b. Approximately 97% of the Japanese cedar’s cells are tracheids. Tracheids in the latewood have thick-walled cells, which consist of the S_2_ layer of 86% in the cedar [54]. The microfibrils of the S_2_ layer are almost fully aligned along the direction of the tracheid [53] and, therefore, its internal structure plays a key role in determining the mechanical properties, especially under applied load parallel to the grain. The microfibril angle (MFA) is the angle between helical windings of microfibrils in the S_2_ layer of the tracheid and the longitudinal cell axis; on research in Sugi, MFA was found to be a crucial factor in obtaining mechanical properties such as stiffness [31,32,33,54,55,56,57] and bending load–deflection behavior [34,58]. A large MFA shows low stiffness, on the other hand, and a small MFA in wood shows high stiffness. In general, each cell was considerably stiffer and stronger parallel to its axis than perpendicular. Therefore, the elastic modulus and tensile strength for the specimen with the tensile fracture were higher than those of the specimen with the shear fracture.

**Figure 13:**
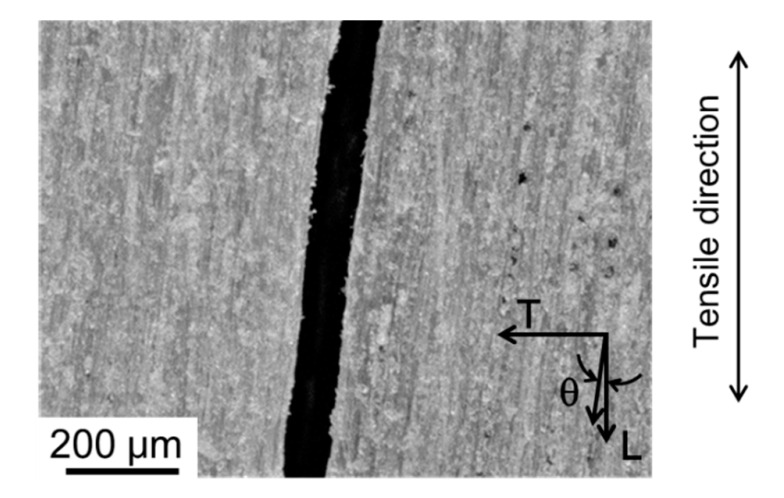
A single shear crack path in the shear fracture observed in the L-T plane.

**Figure 14:**
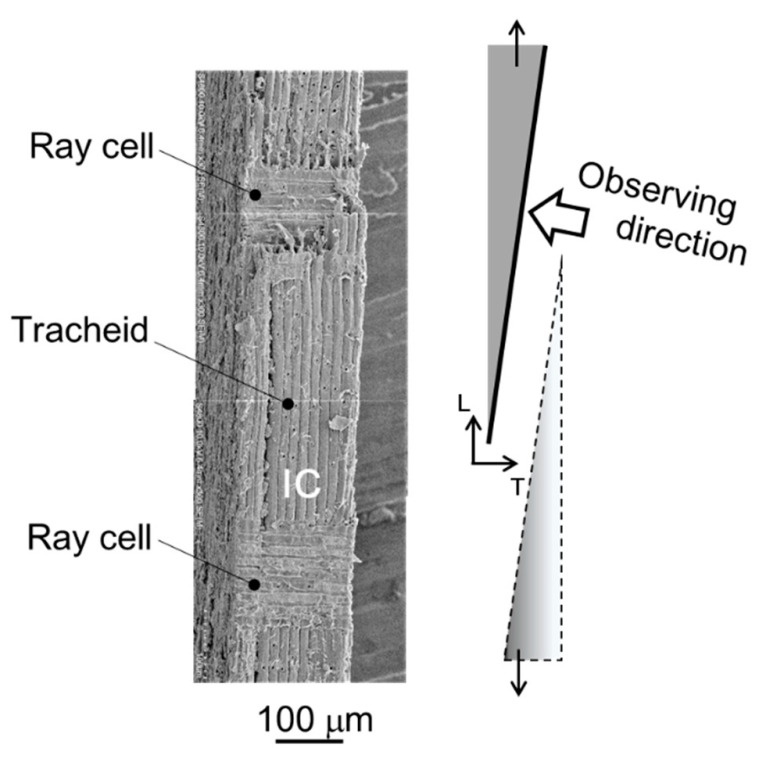
Microfractography of the shear fracture behavior along the tracheid interface and intercellular layer into the ray tissue.

**Figure 15:**
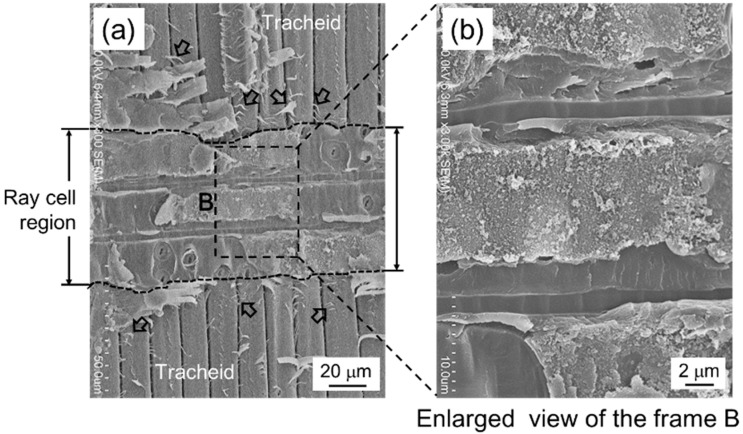
SEM image showing the behavior of the shear fracture. Arrows indicate tearing fibril fragments in the intercell-failed tracheids. The enlarged view (**b**) is section B surrounded by the dashed lines in (**a**).

A photograph of a single shear crack in the shear fracture observed from the L-T plane is shown in Figure 13. Macroscopic shear fractures occurred due to brittleness and at an angle of 12 to 20° in the tensile direction. This corresponds with the growth direction of tracheids being tilted at *θ* of 12 to 20° in the L direction. A microphotograph taken perpendicular to the shear fracture surface is shown in Figure 14. The shear crack propagated through the tracheid interface and intercellular layer into the ray tissue. We confirmed that the crack propagated in a stepwise manner, as shown in the upper part of Figure 14. In addition, the fracture of tracheids was mainly due to interfacial debonding along the lamellar structure of the tracheid. It is known that the dry wood cell interface between tracheids is filled with deposits such as lignin, gum, resin, and tylose [59]. The shear strength was significantly lower than the fibril strength in the tracheid. For this reason, interfacial debonding at the tracheid interface readily occurred. As shown in Figure 4b, the ray tissue configuration in the L-T plane had a high aspect ratio and its tips were sharp. Although the ray tissue and cell structures were not clarified, the shear resistance (i.e., shear modulus and shear strength) of the intercellular layer of ray tissue was weak based on fracture surface observation in this study. Miyoshi et al. measured the breaking length of ray tissue after the lateral tensile test [60] and reported that the mechanical properties of wood in the lateral direction are significantly affected by the structural features such as deformation of cell shapes and arrangement of ray tissue or tracheids [61]. Figure 15 shows another microphotograph of the shear fracture surface. Intercell failure predominated in the entire fracture surface in the tracheid region. Several twisting and tearing fibrils (indicated by arrows) were observed in the region of failed tracheids above and below the microphotograph in Figure 15a. Those of the fibrils were spread out in response to the shear direction, while an open plane without fibrils was viewed in the region of the ray cell (see Figure 15b). The strength characteristics in the region of the ray cell were lower than those of tracheids with fragments spiraling out. The observational finding is obviously evidence to decide the existence of different fracture strength levels on the interface tracheids and ray cell. The crack origin site of the shear fracture in the present study is unknown, but considering the earlier occurrence of shear cracks in latewood, ray cells may be involved. This result suggests that nonlinearity in the stress–strain curve due to shear loading as illustrated in Figure 8 was caused by accumulation fracture at ray cells occurring during testing.

Based on this study, the mechanical properties of Sugi latewood were closely related to the tilt of tracheid grains. Many previous studies demonstrated the influence of the slope of wood grain on the MOE and MOR as follows: Hankinson’s formula is well known as a prediction equation for the strength as a function of the grain angle [62]. Xavier et al. [63] and Bilko et al. [64] reported that grain deviation in the testing force axis causes a degradation in shear strength using the shear arcan test, in which the angle of the grain ranges from 0 to 90°. Gupta et al. revealed the effects of the grain angle on the shear strength by the shear block test [65]. Mania et al. demonstrated that the grain deviation angle has the greatest influence on mechanical parameters, such as elastic energy and work until maximum load, using the bending test with different wood species [66]. Although these studies were conducted using samples containing earlywood and latewood, their conclusions are consistent with the present study in that the tilt of tracheids, *θ*, is important for mechanical properties.

In the present study, although the fracture mechanisms were not clarified in detail, our results suggest that the low mechanical property in ray cells have a distinct effect on fracture morphologies in Sugi latewood. These results might also influence the other properties such as cutting and processing of timber and durability in wood products in large-diameter Sugi logs because GLT and CLT beams are subject to shearing. On the other hand, large-diameter Sugi trees are equivalent to aged Sugi. For effective utilization of Sugi wood resources in the future, it is important to understand the properties of wood derived from aged-Sugi trees.

## 4. Conclusions

In this paper, the tensile stress–strain behavior of latewood as a simple substance specimen collected from the outer side in heartwood in a large-diameter Sugi log was investigated. Based on this study, including tensile examination, Weibull statistics analysis, and fracture surface observation by SEM, we made the following conclusions:

1. Two fracture modes in the L-T plane of latewood, tensile fracture and shear fracture, were revealed by tensile examination. This study suggested that the two fracture modes depend on the tilt of tracheids observed in the L-T plane.
2. The average tensile strength by Weibull statistics analysis of the shear-fractured specimen was approximately 29% lower than that in the tensile-fractured specimen.
3. Fibrils from within the tracheid were closely fully related to tensile fractures in latewood. On the other hand, the shear crack occurred at an angle of 12 to 20° in the tensile direction. There were two features of shear fractures: interfacial debonding of tracheids and crack propagation of the intercellular layer in ray cells. Moreover, it was found as evidence to decide the existence of different fracture strength levels on the interface tracheids and ray cells under shearing.

## Figures and Tables

**Table 1:** Weibull parameters, elastic modulus, *E*, and fracture strain, *ε_f_*_,_ determined by tensile examination in the present study.

	Weibull Parameters					Elastic Modulus, *E*, GPa (Strain Gauge)	Fracture Strain, *ε_f_*, (-)
	*n*	$\sigma_{0},\;\mathrm{MPa}$	*m* Value	Correlation Coefficient, *R*	Mean Tensile Strength, $\sigma_{mean},\;\mathrm{MPa}$		
Tensile fracture	19	180	4.8	0.98	165	13.3	0.015 ± 0.005
Shear fracture	20	126	5.5	0.95	116		0.014 ± 0.006

## Data Availability

Data will be made available upon reasonable request.
